# Deep learning on brain metastasis for predicting EGFR genotype and EGFR-TKI therapy response in metastatic NSCLC: a multicenter study

**DOI:** 10.3389/fbioe.2025.1637095

**Published:** 2025-10-02

**Authors:** Shuailin You, Ying Fan, Zhiguang Yang, Chunna Yang, Yiyao Sun, Yahong Luo, Zekun Wang, Bo Sun, Wenyan Jiang

**Affiliations:** ^1^ College of Technology and Data, Yantai Nanshan University, Yantai, China; ^2^ School of Intelligent Medicine, China Medical University, Shenyang, Liaoning, China; ^3^ College of Biomedical Engineering, Fudan University, Shanghai, China; ^4^ Department of Radiology, Shengjing Hospital, Shenyang, China; ^5^ Department of Medical Imaging, Cancer Hospital of China Medical University, Liaoning Cancer Hospital and Institute, Shenyang, Liaoning, China; ^6^ Department of Radiology, The First Affiliated Hospital of Dalian Medical University, Dalian, Liaoning, China; ^7^ Department of Scientific Research and Academic, Cancer Hospital of China Medical University, Liaoning Cancer Hospital and Institute, Shenyang, Liaoning, China

**Keywords:** NSCLC, EGFR, TKI, brain metastasis, deep learning

## Abstract

**Background:**

Brain metastases are common in patients with advanced non-small cell lung cancer (NSCLC), particularly those harboring EGFR mutations, and accurate prediction of EGFR mutation status and therapeutic response is crucial for guiding targeted therapy. This study aims to conduct a deep learning (DL) approach to automatically predict epidermal growth factor receptor (EGFR) genotype and response to EGFR-tyrosine kinase inhibitor (TKI) therapy in NSCLC patients with brain metastatic tumor (BM).

**Methods:**

For training and validating the DL models, 388 patients were enrolled from three centers between Jul. 2014 and Dec.2022 (230 from center 1, 80 from center 2 and 78 from center 3). Contrast-enhanced T1-weighted (T1CE) and T2-weighted (T2W) brain MRI images before treatment for each patient were obtained for analyses. We developed an EGFR-TKI system (ETS) for automated detection of brain metastatic (BM) lesions and to differentiate EGFR mutation status and predict response to EGFR-TKI therapy. The models underwent rigorous evaluation through receiver operating characteristic (ROC) curve analyses, where metrics such as area under the curve (AUC), sensitivity, and specificity were examined.

**Results:**

For prediction of EGFR mutation status, the ETS integrating radiological-based features and clinical factors achieved AUCs of 0.842, 0.833 and 0.832 on the internal validation, external validation 1 and external validation 2 cohort, respectively. For forecasting response to EGFR-TKI therapy, the fusion model created by amalgamating MRI with clinical factors generated AUCs of 0.747, 0.726 and 0.728 on the internal validation, external validation 1, and external validation 2 cohort, respectively.

**Conclusion:**

The ETS may have the potential to work as a non-invasive tool for predicting EGFR mutation status and response to EGFR-TKI therapy, which holds promise as a non-invasive tool to assist clinicians in making decisions about personalized treatment strategies.

## 1 Introduction

Lung cancer has been a devastating disease and one of the most frequently diagnosed cancers around the world (Sculier, 2013). Lung cancer primarily begins in the lung and may spread to other organs (Boire et al., 2020). The survival statistics of patients with lung cancer are grim, which is often due to the development of distant metastasis (Arbour and Riely, 2019; Schuchert and Luketich, 2003). The brain metastasis (BM) is a major cause of morbidity in lung cancer and frequently results in poor survival rates of less than 1 year (Boire et al., 2020; Niu et al., 2016). And it was reported that approximately half of the lung cancer patients would develop BM (Arbour and Riely, 2019).

Epidermal growth factor receptor (EGFR)-tyrosine kinase inhibitors (TKIs) have been considered as one of the most effective therapeutic strategies for lung cancers (Lynch et al., 2004). Once the patient is diagnosed as an EGFR mutant, EGFR-TKI therapy can be the first-line choice (Yang et al., 2017). However, the effect of the EGFR-TKI is not always satisfactory, and many cases would suffer from tumour progression after receiving the EGFR-TKIs (Rebuzzi et al., 2020). To date, there is still a lack of accurate and reliable methods for the early detection of the EGFR mutation and evaluating therapeutic response to EGFR-TKI before treatment. Although biopsy sampling is routinely used in clinical settings, the biopsy is invasive and may introduce high risks of tissue damage and tumor cell spread (Thompson et al., 2016). In addition, intratumoral heterogeneities can influence the biopsy analysis results because the biopsy can only reflect a limited region in the tumor (Huang W-L. et al., 2017). Therefore, biopsy-based assessment of EGFR mutation status or response to EGFR-TKI is not suggested. Medical imaging-based assessments, on the other hand, are usually subjective and unreliable (Chetan and Gleeson, 2021). Radiologists can hardly evaluate the EGFR mutation status or therapeutic response because of the absence of a specific marker. There is a great need for an effective and non-invasive method to assist in preoperatively determining which patients can benefit from EGFR-TKI therapy.

Radiomics has demonstrated the relationship between underlying biological mechanisms and clinical significance by computing quantitative features directly from medical images (Lambin et al., 2017). While, traditional handcrafted-based radiomics has limitations (Sculier, 2013): handcrafted features are manually calculated based on previously proposed formulas, which can cover only limited types of features (e.g., shape-based, first-order and textural features), and hence result in limited capabilities of digging valuable information from imaging data (Lambin et al., 2017); and (Boire et al., 2020) the process of feature selection and modeling is laborious and time-consuming (Lambin et al., 2017), which cannot be performed as the end-to-end training and testing. In contrast to machine learning-based approaches, deep learning algorithms have been shown to automatically learn representative information from raw data (Pan et al., 2019; Magadza and Viriri, 2021). Deep learning-based models have been proposed for detecting the EGFR mutation, but all focused on thoracic imaging of the primary lung cancer (Wang S. et al., 2019; Yin et al., 2021; Wang et al., 2022). While, clinical evidences have shown that patients with EGFR mutant NSCLC have a high incidence of BM, which is also known as an important indicator to reflect the therapeutic efficacy (Boire et al., 2020; De Cos et al., 2009). Recent handcrafted radiomics studies proved that information highly associated with response to EGFR-TKI can be captured from the NSCLC originated BM (Fan et al., 2023a; Fan et al., 2023b; Fan et al., 2022), but all simply applied conventional machine learning methods on a limited sample size. To our knowledge, there is still no report investigating the value of deep learning in predicting therapeutic efficacy of EGFR-TKI therapy based on BM. In this study, we proposed an automated artificial intelligence EGFR-TKI system (ETS) to predict EGFR genotype and response to EGFR-TKI treatment, aiming to assist clinicians in making appropriate therapeutic plans based on the ETS predicted possibility of obtaining the benefit from EGFR-TKI treatment.

## 2 Methods

### 2.1 Patients

**FIGURE 1:**
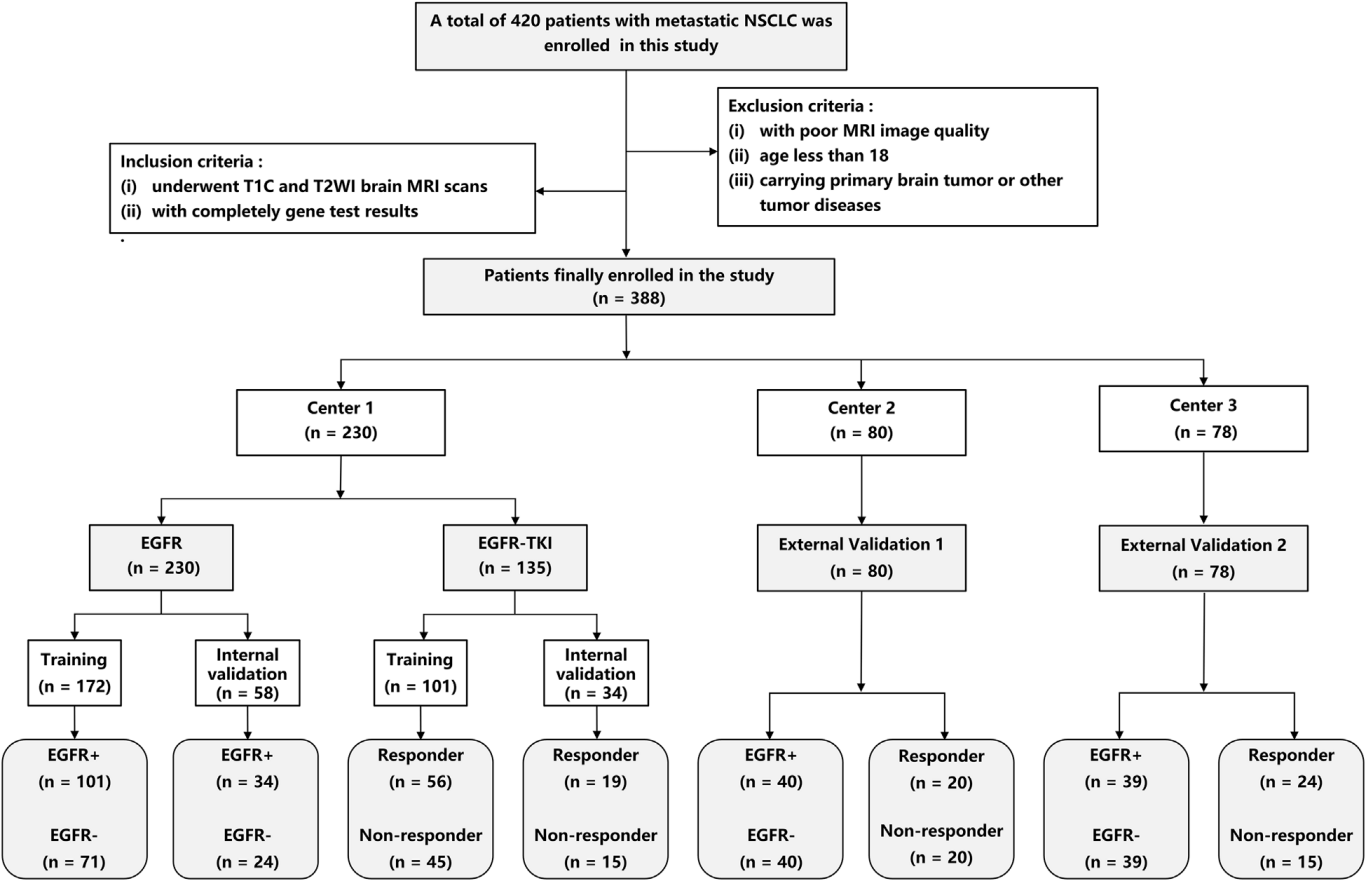
Flowchart of patient recruitment in three centers.

This study was approved by the ethics committee of our hospital. A total of 230 patients were enrolled from center 1 between January 2017 and December 2021 and served as the primary cohort. 80 patients were enrolled from center 2 (between Jul. 2014 and Feb. 2022), and 78 patients were enrolled from center 3 (between Jan. 2020 and Dec. 2022), and served as the external validation cohort 1 and 2, respectively. The Response Evaluation Criteria in Solid Tumors (RECIST) 1.1 (Eisenhauer et al., 2009) was used to determine treatment response to EGFR-TKI therapy. The inclusion criteria include (Sculier, 2013): underwent complete T1CE and T2W brain MRI scans before treatment, and (Boire et al., 2020) had complete gene test results. The exclusion criteria include (Sculier, 2013): with poor MRI image quality (Boire et al., 2020); age less than 18, and (Arbour and Riely, 2019) carrying a primary brain tumor or other tumor diseases. Patients from center 1 were divided into training and internal validation cohorts by random stratified sampling in a ratio of 8:2. Patients from centers 2 and 3 were used as independent sets to validate our DL methods. Figure 1 shows the screening process for patients from all three centers.

### 2.2 MRI acquisition and region of interest (ROI) segmentation

Patients from center 1 were scanned by a 3.0-T MRI scanner (Siemens Verio, Erlangen, Germany), patients from center 2 were scanned by a 3.0-T MRI scanner (Siemens Magnetom Skyra, Erlangen, Germany), and patients from center 3 were scanned by a 3.0-T MRI scanner (Philips, Ingenia). In center 1, the T1CE MRI scanning parameters were as follows: Repeat time (TR) = 270 ms; Echo time (TE) = 2.48 ms; slice thickness = 5 mm, FOV = 194 × 230 mm, and matrix size = 320 × 216. The T2W MRI scanning parameters were as follows: TR = 3630 ms, TE = 87 ms; slice thickness = 5 mm; FOV 194 × 230 mm, and matrix size = 384 × 227 mm. In center 2, the T1CE MRI scanning parameters were as follows: TR = 1400 ms; TE = 9 ms; slice thickness = 6 mm, FOV = 179 × 230 mm and matrix size = 320 × 187. The T2W MRI scanning parameters were as follows: TR = 3500 ms, TE = 99 ms; slice thickness = 6 mm; FOV = 194 × 230 mm and matrix size = 320 × 270 mm. T1CE MRI images were taken 5 min after Gd-DTPA injection. In center 3, the parameters of T1CE and T2W MRI were as follows: T1CE: TR = 180 ms; TE = 2.3 ms; slice thickness = 6 mm, and matrix size = 256 × 256. T2W: TR = 2000 ms; TE = 80 ms; slice thickness = 6 mm, and matrix size = 256 × 256. The dose was 0.2 mL/kg, and the injection speed was 3 mL/s. The segmentation of regions of interest (ROIs) of the brain metastasis (BM) was performed using the ITK-SNAP (version 3.6.1). A radiologist with 5 years’ experience was invited to manually segment the ROI of BM, who was blinded to the clinicopathological information of the patients, except for the tumor location. And a senior radiologist with 15 years’ experience was invited to validate all manual delineations. Volume of peritumoral edema (VPE) was calculated using ITK-SNAP.

### 2.3 Development and validation of the ETS

**FIGURE 2:**
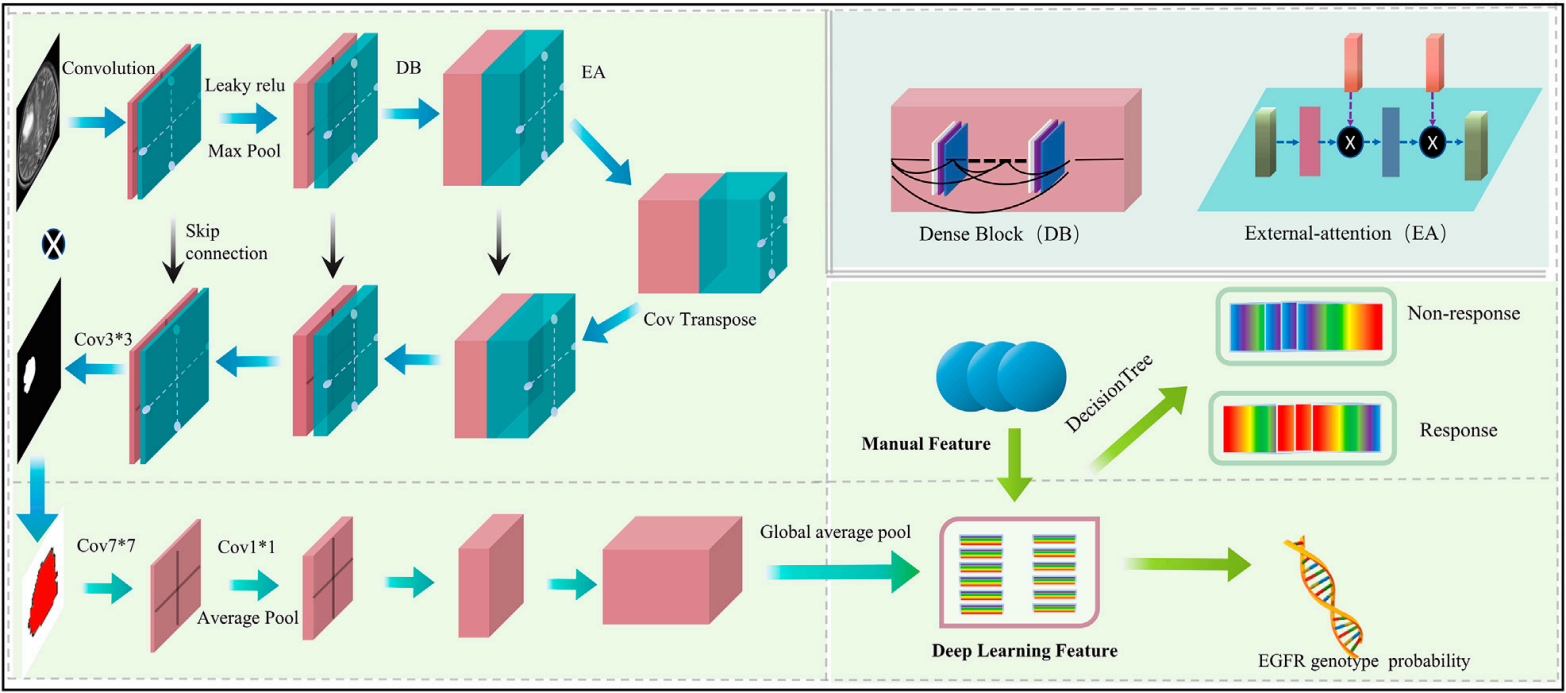
Architecture of the proposed ETS.

The proposed automated artificial intelligence EGFR-TKI system (ETS) consists of two main components: (i) automatic tumor region segmentation and (ii) EGFR genotype prediction. The EGFR-Model of ETS can automatically recognize the region of interest (ROI), and directly predict the EGFR mutation status. For patients with EGFR mutation, the TKI-Model of ETS predicts response to EGFR-TKI therapy. The architecture of the ETS is shown in Figure 2.

The proposed automated artificial intelligence EGFR-TKI system (ETS) consists of two main components: (i) automatic tumor region segmentation and (ii) EGFR genotype prediction. Specifically, ETS first segments the brain metastasis region using a modified FC-DenseNet with LeakyReLU and external attention (EA), and then predicts EGFR mutation status using a DenseNet-121–based classifier. For patients with EGFR mutation, the system further predicts the response to EGFR-TKI therapy. The architecture of ETS is shown in Figure 2.

The segmentation subnetwork for the ETS is based on the FC-Densenet (Jégou et al., 2017) backbone and uses the LeakyRelu nonlinear activation function to replace the ReLU nonlinear activation function. In addition, an EA is added to the network’s downsampling and upsampling process (Guo et al., 2023). To train the segmentation network, we first performed data augmentation to increase the diversity of training samples and improve the robust performance of the training model. Each MRI image is randomly rotated by 90 degrees, and in addition, each image is randomly selected for data enhancement by one of three non-rigid body transformations: Elastic transform, Grid distortion, and Optical distortion. In the training process, the model is optimally trained by adaptive moment estimation (Adam) (Kinga and Adam, 2015) with a learning rate of 0.0001, the total number of iterations of the training model is 100, and the input size of the model is 128 × 128 × 3.

The classification subnetwork uses the Densenet-121 (Huang G. et al., 2017) as the backbone network. The fully connected layer of the Densenet-121 was replaced with the global average pooling (GAP) (Lin et al., 2025) for discriminating the EGFR mutation status. We applied the ideology of transfer learning, where the classification network was pre-trained on the ImageNet-1k dataset to increase the learning efficiency of the network. We evaluated four model variants, No Seg–VPE, No VPE, No Seg, and Seg–VPE—to isolate the contributions of the segmentation network and the volumetric peritumoral edema (VPE) feature.

To predict EGFR-TKI therapy response, we extracted DL features and handcrafted features from patients with EGFR mutation. The analysis of variance (ANOVA) and principal component analysis (PCA) (Witten et al., 2013) were applied to dimensionality reduction and screen features. Finally, we used a decision tree model to predict the response to EGFR-tyrosine TKI therapy. To enhance interpretability and reveal spatial correlations between image regions and prediction results, we applied Grad-CAM (Selvaraju et al., 2017) to the final convolutional layer of the DenseNet-121 classifier. This allowed us to visualize the discriminative regions that most influenced the EGFR mutation prediction. Since the classifier receives input features extracted from the segmented tumor region, the resulting attention maps reflect localized regions within the BM that are most relevant to the model’s decision-making process. In the training process, the model is optimally trained by adaptive moment estimation (Adam) (Kinga and Adam, 2015) with a learning rate of 0.0001; the epoch of the training model was set to 100. All DL experiments were performed in Python (v.3.6) using Keras (version 2.3) on a single GPU (Nvidia GeForce 3090) workstation.

To validate the predictive performance of the ETS for both EGFR‐mutation status and EGFR‐TKI response, we conducted independent evaluations on three datasets: an internal hold-out set (20% of the development data) and two external validation cohorts. The fully trained ETS was applied to each dataset. For each task and each cohort (Internal Validation, External Validation 1, External Validation 2), we generated receiver operating characteristic (ROC) curves and calculated the area under the curve (AUC), accuracy, F1 score, precision, and recall. Optimal decision thresholds were selected by maximizing Youden’s index.

### 2.4 Statistical analysis

**FIGURE 3:**
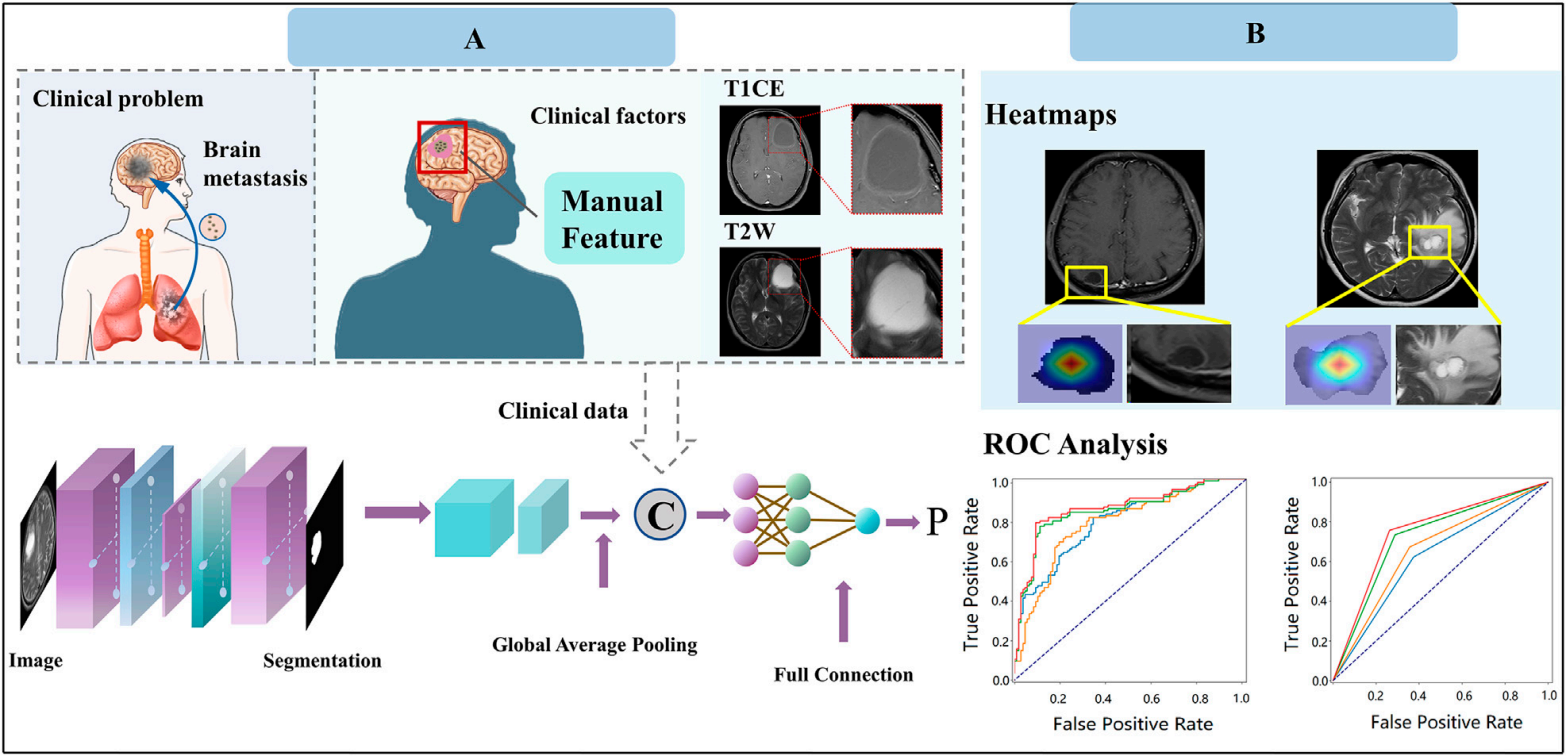
Study design of our work for predicting response to EGFR-TKI treatment. **(A)** Model construction. **(B)** Model application.

All statistical analysis was performed in R software (version 3.6.0). ANOVA was performed for continuous variables, and the chi-square test was used for discrete (categorical) variables. Factors with a p-value less than 0.05 were considered statistically significant. The performance of the ETS was evaluated using area under the curve (AUC), accuracy, F1 score, precision, and recall. All evaluation metrics were implemented in Python (v.3.6) using the scikit-learn library. The Gradient Weighted Class Activation Map (Grad-CAM) was implemented on PyTorch (Version 1.12.0). Figure 3 depicts the workflow of our study.

## 3 Results

### 3.1 Clinical characteristics

Table 1 listed demographic and clinical characteristics of the patients with BM originated from primary NSCLC. From Table 1, there was no statistical significance in terms of age, gender, and smoking history.

**TABLE 1:** Clinical characteristic of patients from three centers.

Characteristic	Center1 (n = 230)	Center 2 (n = 80)	Center 3 (n = 78)	*P*
Age (Mean ± SD)	58.52 ± 9.64	57 ± 10.3	62.26 ± 9	0.216
Sex				0.276
Male	102 (44.3%)	44 (55.0%)	42 (53.8%)	
Female	128 (55.7%)	36 (45.0%)	36 (46.2%)	
Smoking History				0.137
Yes	92 (40.0%)	26 (32.5%)	30 (38.5%)	
No	138 (60.0%)	54 (67.5%)	48 (61.5%)	
PS Score				<0.001
<2	144 (62.6%)	75 (93.75%)	67 (85.9%)	
≥2	86 (37.4%)	5 (6.25%)	11 (14.1%)	

SD, standard deviation; PS, performance status.

### 3.2 Performance for predicting EGFR mutation status

**FIGURE 4:**
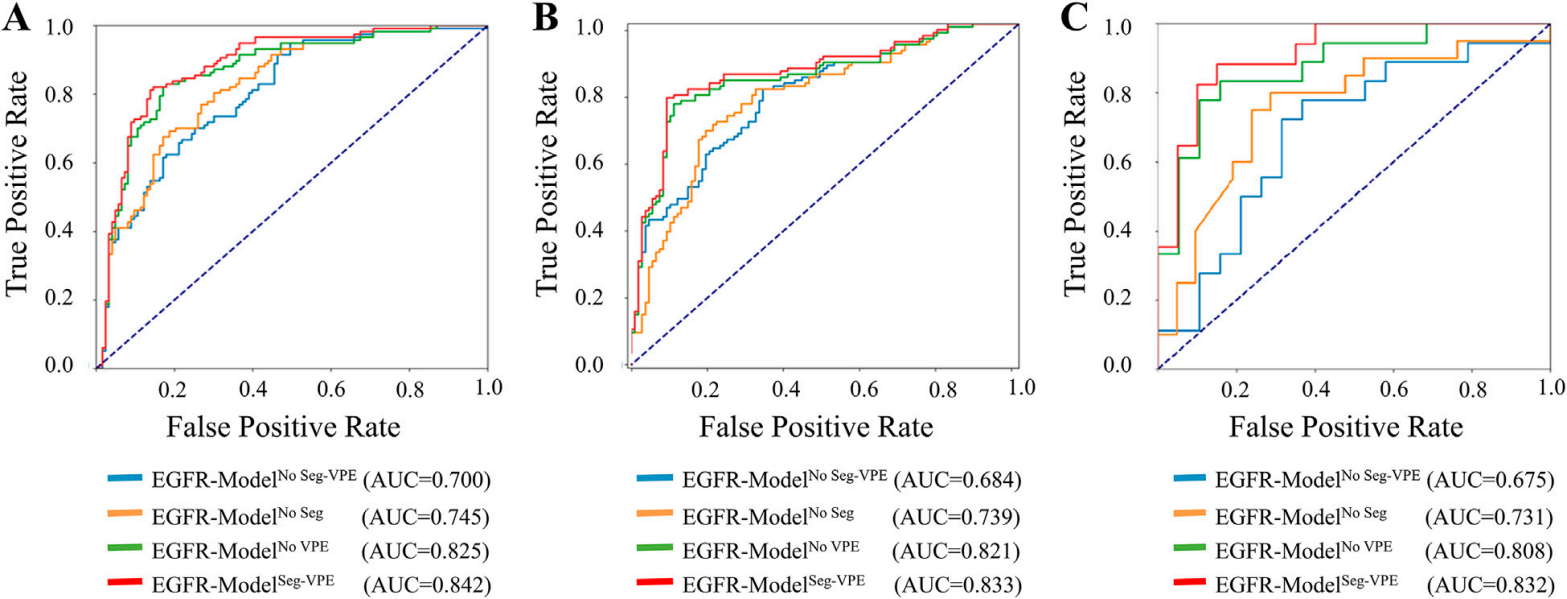
ROC curves of the ETS for predicting the EGFR mutation status in the internal validation **(A)**, external validation 1 **(B)**, and external validation 2 **(C)** set.

**FIGURE 5:**
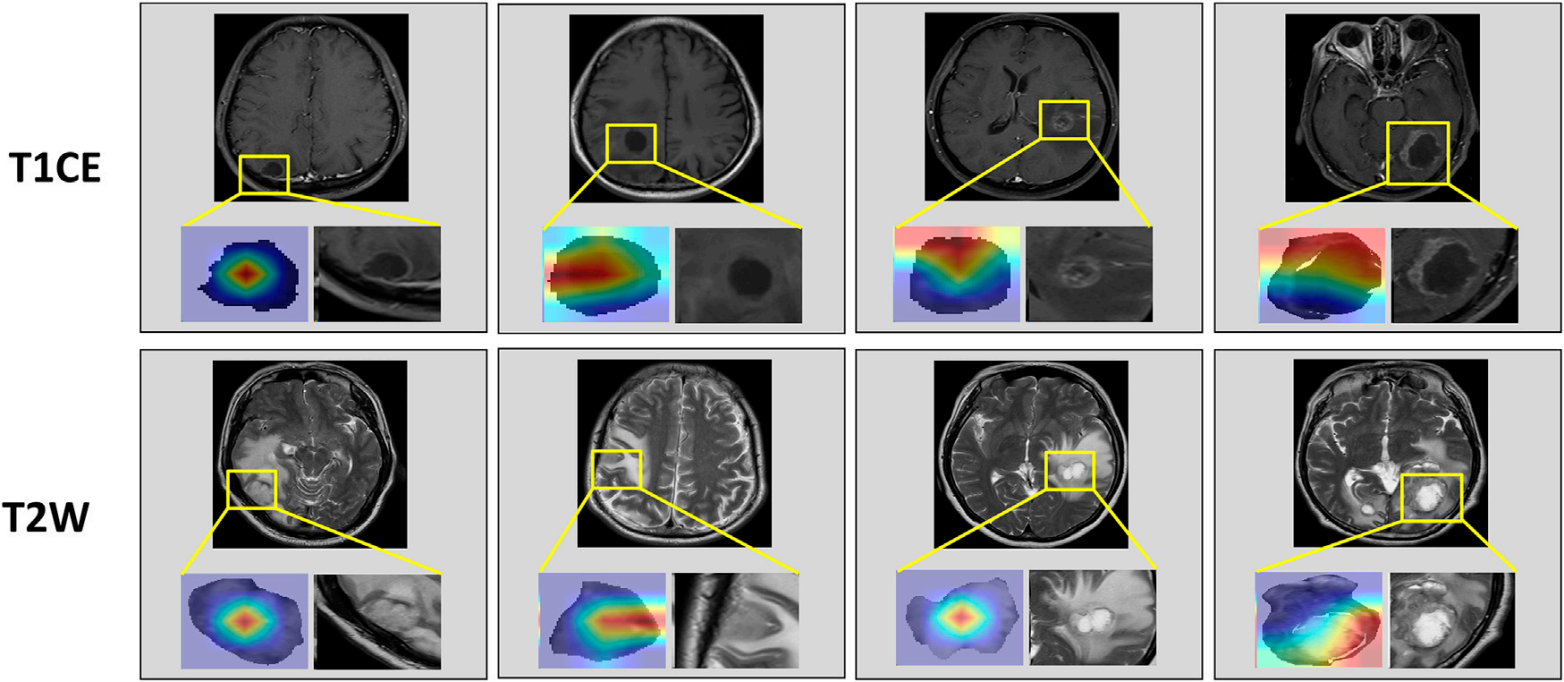
Attention heatmaps on the brain metastasis (BM) visualized by Grad-CAM. The first row shows heatmaps in T1CE MRI. The second row shows heatmaps in T2W MRI.

Table 2 compared the performance of the proposed EGFR-Model^No Seg−VPE^, EGFR-Model^No VPE^, EGFR-Model^No Seg^ and EGFR-Model^Seg-VPE^ for predicting the EGFR mutation status. Without the subnetwork for segmentating the BM, the EGFR-Model^No Seg−VPE^ yielded lower AUCs, accuracy, F1-score, precision, and recall compared with EGFR-Model^No VPE^ in primary and external cohorts. The decreased predictive performance in EGFR-Model^No Seg−VPE^ suggested the necessity of the segmentation subnetwork. By integrating VPE, the EGFR-Model^No Seg^ showed better performance than EGFR-Model^No Seg−VPE^ in terms of AUC, accuracy, F1-score, precision, and recall. This indicated that the VPE can provide additional information to improve the capability of predicting the EGFR mutation status. The EGFR-Model^Seg-VPE^, integrating both VPE and segmentation subnetworks, performed the best among all models for predicting the EGFR mutation status. ROC curves of all models on primary and external sets were shown in Figure 4. As shown in Figure 5, the Grad-CAM heatmaps highlight high-response areas within the segmented tumor region, indicating that the prediction of EGFR mutation status is driven by biologically relevant features. These results illustrate a link between the model architecture, particularly the segmentation-guided feature extraction, and the spatial mapping of predictive regions.

**TABLE 2:** Performance of the ETS for predicting the EGFR mutation status.

Model	Cohort	AUC	Accuracy	F1-score	Precision	Recall
EGFR-Model^No Seg−VPE^	Internal Validation	0.700	0.699	0.694	0.720	0.664
	External Validation 1	0.684	0.683	0.696	0.654	0.626
	External Validation 2	0.675	0.676	0.675	0.675	0.675
EGFR-Model^No Seg^	Internal Validation	0.745	0.743	0.734	0.784	0.690
	External Validation 1	0.739	0.738	0.751	0.699	0.812
	External Validation 2	0.731	0.732	0.731	0,732	0.731
EGFR-Model^No VPE^	Internal Validation	0.825	0.823	0.871	0.873	0.767
	External Validation 1	0.821	0.821	0.819	0.808	0.829
	External Validation 2	0.808	0.811	0.809	0.820	0.808
EGFR-Model^Seg-VPE^	Internal Validation	0.842	0.841	0.835	0.892	0.784
	External Validation 1	0.833	0.833	0.828	0.835	0.821
	External Validation 2	0.832	0.838	0.835	0.842	0.832

EGFR-Model^No Seg−VPE^: Without the subnetwork for segmentation and without adding volume of peritumoral edema (VPE); EGFR-Model^No Seg^.

Without the subnetwork for segmentation; EGFR-Model^No VPE^: Without adding VPE; EGFR-Model^Seg-Vpe^: combined subnetwork for segmentation and VPE.

### 3.3 Performance for predicting response to EGFR-TKI therapy

**FIGURE 6:**
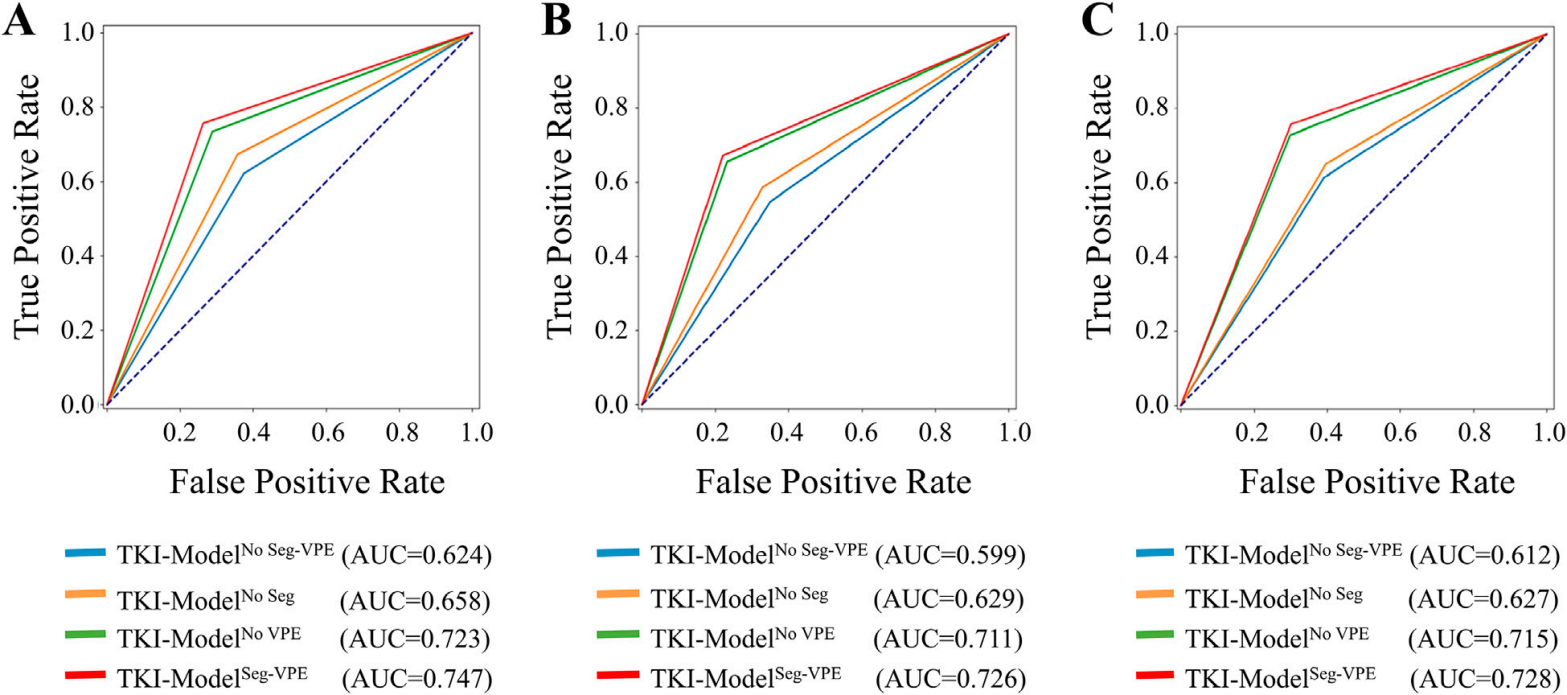
ROC curves of the ETS for predicting response to EGFR-TKI in the internal validation **(A)**, external validation 1 **(B)**, and external validation 2 **(C)** set.

Table 3 compared the performance of the proposed TKI-Model^No Seg−VPE^, TKI-Model^No VPE^, TKI-Model^No Seg^ and TKI-Model^Seg-VPE^ for predicting response to EGFR-TKI. The TKI-Model^No Seg−VPE^ without the subnetwork for segmenting the BM genarated lower AUC and ACC compared with TKI-Model^No VPE^ that has the segmentation subnetwork. The result indicates the necessity of the segmentation subnetwork. The TKI-Model^No Seg^ integrating VPE outperformed the TKI-Model^No Seg−VPE^ that is without VPE in terms of AUC and ACC in primary and external cohorts. This suggested that the VPE holds additional information correlated to the efficacy of EGFR-TKI. The TKI-Model^Seg-VPE^ integrating both VPE and the segmentation subnetwork achieved the best predictive performance with AUCs of 0.747, 0.726, and 0.728 in the internal validation, external validation 1 and external validation 2 cohort, respectively. Figure 6 depicted the ROC curves of the TKI-Model for predicting response to EGFR-TKI.

**TABLE 3:** Performance of the TKI-Model for predicting response to EGFR-TKI.

Model	Cohort	AUC	Accuracy	F1-score	Precision	Recall
TKI-Model^No Seg−VPE^	Internal Validation	0.624	0.624	0.636	0.651	0.622
	External Validation 1	0.599	0.603	0.558	0.569	0.547
	External Validation 2	0.612	0.612	0.612	0.612	0.612
TKI-Model^No Seg^	Internal Validation	0.658	0.660	0.686	0.700	0.673
	External Validation 1	0.629	0.632	0.672	0.652	0.662
	External Validation 2	0.627	0.629	0.627	0.627	0.627
TKI-Model^No VPE^	Internal Validation	0.723	0.724	0.746	0.758	0.734
	External Validation 1	0.711	0.711	0.697	0.745	0.655
	External Validation 2	0.715	0.717	0.714	0.714	0.715
TKI-Model^Seg-VPE^	Internal Validation	0.747	0.748	0.768	0.779	0.757
	External Validation 1	0.726	0.725	0.713	0.759	0.672
	External Validation 2	0.728	0.733	0.727	0.726	0.729

TKI-Model^No Seg−VPE^: Without the subnetwork for segmentation and without adding volume of peritumoral edema (VPE); TKI-Model^No Seg^.

Without the subnetwork for segmentation; TKI-Model^No VPE^: Without adding VPE; TKI -Model^Seg-Vpe^: combined subnetwork for segmentation and VPE.

## 4 Discussion

Current guidelines for clinical assessment of EGFR genotype and therapeutic response to EGFR-TKI rely on visual radiologic assessment, which is subjectively biased and unreliable (Lowery and Yu, 2017). Previous works have shown the power of deep learning in evaluating the efficiency of EGFR-TKI treatment in lung cancer (Song et al., 2021; Deng et al., 2022; Lu et al., 2023), but all have been based on the primary lesion. To our knowledge, deep learning has not been applied to lung cancer-originated brain metastasis (BM) for determining the presence of EGFR mutation and the efficiency of EGFR-TKI therapy.

This study constructed an ETS integrating a segmentation subnetwork and a classification subnetwork. Considering the BM only occupies a small percentage of the brain area, and thus using the whole brain MRI image as input to the network may introduce numerous noise features, we extracted the BM as an upstream task to determine the EGFR genotype. Prior research has indicated that lesion size plays a pivotal role in segmentation accuracy (Wang F. et al., 2019). To enhance the efficiency and expediency of brain tumor extraction, we expanded the region of interest (ROI) by 5 pixels to create a mask patch, thereby increasing the area of the segmentation region. Meanwhile, the external attention (Guo et al., 2023) was introduced into our segmentation subnetwork, which implicitly considers the relationship between different brain MRI feature maps and weights, and sums the different feature maps to realize the effective fusion of information, thus improving the segmentation performance.

Our classification network conducts feature extraction on the patch, including BM. Concurrently, handcrafted features are introduced to augment the comprehensiveness of the features, thereby enhancing the accuracy of EGFR prediction. This approach aligns, in part, with the findings by Nanni et al. (2017), which underscored the contribution of manual features in improving classification accuracy. Our model underwent a more detailed analysis based on both deep learning features and handcrafted features. The developed EGFR-Model generated AUCs of 0.832, 0.833, and 0.842 for predicting the EGFR mutation in the internal validation, external validation 1, and external validation 2 sets, respectively. This was much higher than previous works based on the primary lesion that obtained AUCs ranging from 0.575 to 0.762 (Tu et al., 2019; Mei et al., 2018; Digumarthy et al., 2019; Liu et al., 2016; Zhang et al., 2018; Gevaert et al., 2017; Yuan et al., 2017; Pinheiro et al., 2020). Our TKI-Model also outperformed the recent handcrafted-based radiomics study based on BM that generated AUCs ranging from 0.671 to 0.780 (Wang et al., 2021). The model’s effectiveness was further validated using a decision tree applied to both deep learning and handcrafted features. This dual-pronged approach showcased the model’s robust performance in predicting EGFR genotypes and treatment efficacy. The concurrent demonstration of efficacy on the internal validation set and two external test sets attests to the strong generalization ability of our model, as presented in Table 2, 3. This underscores its potential as a versatile tool for clinical decision-making in the context of personalized treatment for NSCLC patients with BM.

We identified the volume of peritumoral edema (VPE) as an independent clinical factor that is highly associated with the EGFR mutation status and response to EGFR-TKI. Integration of the VPE to the ETS can improve the system’s performance. The finding is consistent with previous histopathological reports that indicated that the peritumoral edema is causally linked to compressive ischemia, vascular shunting attributable to membranous microvascular parasitism, and secretory-excretory phenomena within tumor cells (Tamiya et al., 2001; Nakasu et al., 2005). Moreover, the cortical blood supply emerges as a critical factor influencing the development of peritumoral edema (Tamiya et al., 2001; Nakasu et al., 2005). This insight underscores the multifaceted nature of peritumoral edema and its relevance as a clinically significant factor in predicting EGFR mutation and response to EGFR-TKI. Our finding was supported by recent radiomics studies focusing on primary brain tumors that showed the peritumoral edema holds additional information associated with tumor diagnoses beyond the primary lesion (Kim et al., 2018; Prasanna et al., 2017; Joo et al., 2021), and the VPE and imaging-based radiomics can provide complementary information (Fan et al., 2023a).

First, the current study was retrospective, and the developed models therefore need to be further validated with prospective data. Second, the study only evaluated T1CE and T2W MRI, and the performance of the models may be potentially improved by incorporating more MRI sequences, e.g., diffusion-weighted imaging and fluid-attenuated inversion recovery MRI. Third, it is pivotal to recognize that the segmentation network used in this study operates at a patch level. For a more meticulous delineation of tumor boundaries, there exists a need for a segmentation approach that offers greater precision.Fourth, this study focused on predicting the presence of EGFR mutation, without differentiating specific subtypes such as exon 19 deletion or L858R. This may limit the model’s utility for precise therapeutic decision-making. Future work will explore subtype-level prediction for improved clinical relevance. Finally, this study only evaluated the EGFR gene mutation; other important genes that may also influence the effect of targeted therapy should be included in future studies.

## 5 Conclusion

In this study, we developed an automated EGFR-TKI system (ETS) to detect brain metastases and predict EGFR mutation status and therapy response.The system has been validated in both internal and external cohorts, demonstrating consistent performance. As a non-invasive method for detecting EGFR mutations, it holds potential to assist clinical decision-making and provide valuable support for non-small cell lung cancer (NSCLC) patients undergoing EGFR-TKI treatment.

## Data Availability

The datasets presented in this article are not readily available due to ethical restrictions involving patient privacy and hospital regulations. Requests to access the datasets should be directed to Wenyan Jiang xiaoya83921@163.com.

## References

[B1] Arbour K. C. Riely G. J. (2019). Systemic therapy for locally advanced and metastatic non-small cell lung cancer A review. Jama-J Am. Med. Assoc. 322 (8), 764–774. 10.1001/jama.2019.11058 31454018

[B2] Boire A. Brastianos P. K. Garzia L. Valiente M. (2020). Brain metastasis. Nat. Rev. Cancer 20 (1), 4–11. 10.1038/s41568-019-0220-y 31780784

[B3] Chetan M. R. Gleeson F. V. (2021). Radiomics in predicting treatment response in non-small-cell lung cancer: current status, challenges and future perspectives. Eur. Radiol. 31, 1049–1058. 10.1007/s00330-020-07141-9 32809167 PMC7813733

[B4] de Cos J. S. González M. A. S. Montero M. V. Calvo M. C. P. Vicente M. J. M. Valle M. H. (2009). Non-small cell lung cancer and silent brain metastasis. Lung Cancer 63 (1), 140–145. 10.1016/j.lungcan.2008.04.013 18556086

[B5] Deng K. Wang L. Liu Y. Li X. Hou Q. Cao M. (2022). A deep learning-based system for survival benefit prediction of tyrosine kinase inhibitors and immune checkpoint inhibitors in stage IV non-small cell lung cancer patients: a multicenter, prognostic study. EClinicalMedicine 51, 101541. 10.1016/j.eclinm.2022.101541 35813093 PMC9256845

[B6] Digumarthy S. R. Padole A. M. Lo Gullo R. Sequist L. V. Kalra M. K. (2019). Can CT radiomic analysis in NSCLC predict histology and EGFR mutation status? Medicine 98 (1), e13963. 10.1097/md.0000000000013963 30608433 PMC6344142

[B7] Eisenhauer E. A. Therasse P. Bogaerts J. Schwartz L. Sargent D. Ford R. (2009). New response evaluation criteria in solid tumours: revised RECIST guideline (version 1.1). Eur. J. cancer 45 (2), 228–247. 10.1016/j.ejca.2008.10.026 19097774

[B8] Fan Y. Zhao Z. L. Wang X. L. Ai H. Yang C. Luo Y. (2022). Radiomics for prediction of response to EGFR-TKI based on metastasis/brain parenchyma (M/BP)-interface. Radiol. Med. 127 (12), 1342–1354. 10.1007/s11547-022-01569-3 36284030

[B9] Fan Y. Wang X. Yang C. Chen H. Wang H. Wang X. (2023a). Brain‐tumor interface‐based MRI radiomics models to determine EGFR mutation, response to EGFR‐TKI and T790M resistance mutation in non‐small cell lung carcinoma brain metastasis. J. Magnetic Reson. Imaging 58, 1838–1847. 10.1002/jmri.28751 37144750

[B10] Fan Y. Wang X. T. Dong Y. Cui E. Wang H. Sun X. (2023b). Multiregional radiomics of brain metastasis can predict response to EGFR-TKI in metastatic NSCLC. Eur. Radiol. 33, 7902–7912. 10.1007/s00330-023-09709-7 37142868

[B11] Gevaert O. Echegaray S. Khuong A. Hoang C. D. Shrager J. B. Jensen K. C. (2017). Predictive radiogenomics modeling of EGFR mutation status in lung cancer. Sci. Rep-Uk 7, 41674. 10.1038/srep41674 28139704 PMC5282551

[B12] Guo M. H. Liu Z. N. Mu T. J. Hu S. M. (2023). Beyond self-attention: external attention using two linear layers for visual tasks. Ieee Trans. Pattern Analysis Mach. Intell. 45 (5), 5436–5447. 10.1109/TPAMI.2022.3211006 36197869

[B13] Huang W.-L. Chen Y.-L. Yang S.-C. Ho C. L. Wei F. Wong D. T. (2017). Liquid biopsy genotyping in lung cancer: ready for clinical utility? Oncotarget 8 (11), 18590–18608. 10.18632/oncotarget.14613 28099915 PMC5392351

[B14] Huang G. Liu Z. Van Der Maaten L. Weinberger K. Q. (2017). “Densely connected convolutional networks,” in Proceedings of the IEEE conference on computer vision and pattern recognition, 4700–4708.

[B15] Jégou S. Drozdzal M. Vazquez D. Romero A. Bengio Y. (2017). “The one hundred layers tiramisu: fully convolutional densenets for semantic segmentation,” in Proceedings of the IEEE conference on computer vision and pattern recognition workshops, 11–19.

[B16] Joo L. Park J. E. Park S. Y. Nam S. J. Kim Y. H. Kim J. H. (2021). Extensive peritumoral edema and brain-to-tumor interface MRI features enable prediction of brain invasion in meningioma: development and validation. Neuro-Oncology 23 (2), 324–333. 10.1093/neuonc/noaa190 32789495 PMC8631067

[B17] Kinga D. Adam J. B. (2015). “A method for stochastic optimization,”Int. Conf. Learn. Represent. (ICLR) 5 6. Available online at: https://arxiv.org/pdf/1412.6980

[B18] Kim Y. Cho H.-h. Kim S. T. Park H. Nam D. Kong D.-S. (2018). Radiomics features to distinguish glioblastoma from primary central nervous system lymphoma on multi-parametric MRI. Neuroradiology 60, 1297–1305. 10.1007/s00234-018-2091-4 30232517

[B19] Lambin P. Leijenaar R. T. H. Deist T. M. Peerlings J. de Jong E. E. van Timmeren J. (2017). Radiomics: the bridge between medical imaging and personalized medicine. Nat. Rev. Clin. Oncol. 14 (12), 749–762. 10.1038/nrclinonc.2017.141 28975929

[B20] Lin M. Chen Q. Yan S. (2025). Network in network. arXiv preprint arXiv:13124400 2013.

[B21] Liu Y. Kim J. Balagurunathan Y. Li Q. Garcia A. L. Stringfield O. (2016). Radiomic features are associated with EGFR mutation status in lung adenocarcinomas. Clin. Lung Cancer 17 (5), 441–448.e6. 10.1016/j.cllc.2016.02.001 27017476 PMC5548419

[B22] Lowery F. J. Yu D. H. (2017). Brain metastasis: unique challenges and open opportunities. Bba-Rev Cancer 1867 (1), 49–57. 10.1016/j.bbcan.2016.12.001 27939792 PMC5272787

[B23] Lu C. F. Liao C. Y. Chao H. S. Chiu H. Y. Wang T. W. Lee Y. (2023). A radiomics-based deep learning approach to predict progression free-survival after tyrosine kinase inhibitor therapy in non-small cell lung cancer. Cancer Imaging 23 (1), 9. 10.1186/s40644-023-00522-5 36670497 PMC9854198

[B24] Lynch T. J. Bell D. W. Sordella R. Gurubhagavatula S. Okimoto R. A. Brannigan B. W. (2004). Activating mutations in the epidermal growth factor receptor underlying responsiveness of non-small-cell lung cancer to gefitinib. New Engl. J. Med. 350 (21), 2129–2139. 10.1056/nejmoa040938 15118073

[B25] Magadza T. Viriri S. (2021). Deep learning for brain tumor segmentation: a survey of state-of-the-art. J. Imaging 7 (2), 19. 10.3390/jimaging7020019 34460618 PMC8321266

[B26] Mei D. D. Luo Y. Wang Y. Gong J. S. (2018). CT texture analysis of lung adenocarcinoma: can Radiomic features be surrogate biomarkers for EGFR mutation statuses. Cancer Imaging 18, 52. 10.1186/s40644-018-0184-2 30547844 PMC6295009

[B27] Nakasu S. Fukami T. Jito J. Matsuda M. (2005). Microscopic anatomy of the brain–meningioma interface. Brain Tumor Pathol. 22, 53–57. 10.1007/s10014-005-0187-0 18095106

[B28] Nanni L. Ghidoni S. Brahnam S. (2017). Handcrafted vs. non-handcrafted features for computer vision classification. Pattern Recogn. 71, 158–172. 10.1016/j.patcog.2017.05.025

[B29] Niu F.-Y. Zhou Q. Yang J.-J. Zhong W. Z. Chen Z. H. Deng W. (2016). Distribution and prognosis of uncommon metastases from non-small cell lung cancer. BMC cancer 16, 149–6. 10.1186/s12885-016-2169-5 26911831 PMC4766662

[B30] Pan C. C. Schoppe O. Parra-Damas A. Cai R. Todorov M. I. Gondi G. (2019). Deep learning reveals cancer metastasis and therapeutic antibody targeting in the entire body. Cell 179 (7), 1661–1676.e19. 10.1016/j.cell.2019.11.013 31835038 PMC7591821

[B31] Pinheiro G. Pereira T. Dias C. Freitas C. Hespanhol V. Costa J. L. (2020). Identifying relationships between imaging phenotypes and lung cancer-related mutation status: EGFR and KRAS. Sci. Rep-Uk 10 (1), 3625. 10.1038/s41598-020-60202-3 32107398 PMC7046701

[B32] Prasanna P. Patel J. Partovi S. Madabhushi A. Tiwari P. (2017). Radiomic features from the peritumoral brain parenchyma on treatment-naive multi-parametric MR imaging predict long versus short-term survival in glioblastoma multiforme: preliminary findings. Eur. Radiol. 27, 4188–4197. 10.1007/s00330-016-4637-3 27778090 PMC5403632

[B33] Rebuzzi S. E. Alfieri R. La Monica S. Minari R. Petronini P. G. Tiseo M. (2020). Combination of EGFR-TKIs and chemotherapy in advanced EGFR mutated NSCLC: review of the literature and future perspectives. Crit. Rev. Oncology/Hematology 146, 102820. 10.1016/j.critrevonc.2019.102820 31785991

[B34] Schuchert M. J. Luketich J. D. (2003). Solitary sites of metastatic disease in non-small cell lung cancer. Curr. Treat. options Oncol. 4, 65–79. 10.1007/s11864-003-0033-8 12525281

[B35] Sculier J.-P. (2013). Nonsmall cell lung cancer. Eur. Respir. Rev. 22 (127), 33–36. 10.1183/09059180.00007012 23457162 PMC9487433

[B36] Selvaraju R. R. Cogswell M. Das A. Vedantam R. Parikh D. Batra D. (2017). “Grad-cam: visual explanations from deep networks via gradient-based localization,” in Proceedings of the IEEE international conference on computer vision, 618–626. 10.1109/iccv.2017.74

[B37] Song J. Wang L. Ng N. N. (2021). An opportunity to reduce disparities in lung cancer screening. Jama Netw. Open 4 (2), e2129126. 10.1001/jamanetworkopen.2021.29126 34636918

[B38] Tamiya T. Ono Y. Matsumoto K. Ohmoto T. (2001). Peritumoral brain edema in intracranial meningiomas: effects of radiological and histological factors. Neurosurgery 49 (5), 1046–1052. 10.1227/00006123-200111000-00003 11846896

[B39] Thompson J. C. Yee S. S. Troxel A. B. Savitch S. L. Fan R. Balli D. (2016). Detection of therapeutically targetable driver and resistance mutations in lung cancer patients by next-generation sequencing of cell-free circulating tumor DNA. Clin. Cancer Res. 22 (23), 5772–5782. 10.1158/1078-0432.ccr-16-1231 27601595 PMC5448134

[B40] Tu W. T. Sun G. Y. Fan L. Wang Y. Xia Y. Guan Y. (2019). Radiomics signature: a potential and incremental predictor for EGFR mutation status in NSCLC patients, comparison with CT morphology. Lung Cancer 132, 28–35. 10.1016/j.lungcan.2019.03.025 31097090

[B41] Wang S. Shi J. Y. Ye Z. X. Dong D. Yu D. Zhou M. (2019). Predicting EGFR mutation status in lung adenocarcinoma on computed tomography image using deep learning. Eur. Respir. J. 53 (3), 1800986. 10.1183/13993003.00986-2018 30635290 PMC6437603

[B42] Wang F. Jiang R. Zheng L. Meng C. Biswal B. (2019). “3d u-net based brain tumor segmentation and survival days prediction,” in International MICCAI brainlesion workshop (Springer), 131–141.

[B43] Wang G. Y. Wang B. M. Wang Z. Li W. Xiu J. Liu Z. (2021). Radiomics signature of brain metastasis: prediction of EGFR mutation status. Eur. Radiol. 31 (7), 4538–4547. 10.1007/s00330-020-07614-x 33439315

[B44] Wang S. Yu H. Gan Y. C. Wu Z. Li E. Li X. (2022). Mining whole-lung information by artificial intelligence for predicting EGFR genotype and targeted therapy response in lung cancer: a multicohort study. Lancet Digit. Health 4 (5), E309–E319. 10.1016/s2589-7500(22)00024-3 35341713

[B45] Witten D. James G. Hastie T. Tibshirani R. (2013). An introduction to statistical learning with applications in R. New York, NY: springer publication.

[B46] Yang J. J. Zhou C. C. Huang Y. S. Feng J. Lu S. Song Y. (2017). Icotinib versus whole-brain irradiation in patients with EGFR-mutant non-small-cell lung cancer and multiple brain metastases (BRAIN): a multicentre, phase 3, open-label, parallel, randomised controlled trial. Lancet Resp. Med. 5 (9), 707–716. 10.1016/s2213-2600(17)30262-x 28734822

[B47] Yin G. Wang Z. Song Y. Li X. Chen Y. Zhu L. (2021). Prediction of EGFR mutation status based on 18F-FDG PET/CT imaging using deep learning-based model in lung adenocarcinoma. Front. Oncol. 11, 709137. 10.3389/fonc.2021.709137 34367993 PMC8340023

[B48] Yuan M. Pu X. H. Xu X. Q. Zhang Y. D. Zhong Y. Li H. (2017). Lung adenocarcinoma: assessment of epidermal growth factor receptor mutation status based on extended models of diffusion-weighted image. J. Magnetic Reson. Imaging 46 (1), 281–289. 10.1002/jmri.25572 28054731

[B49] Zhang L. W. Chen B. J. Liu X. Song J. Fang M. Hu C. (2018). Quantitative biomarkers for prediction of epidermal growth factor receptor mutation in non-small cell lung cancer. Transl. Oncol. 11 (1), 94–101. 10.1016/j.tranon.2017.10.012 29216508 PMC6002350

